# Altered T-Lymphocyte Biology Following High-Dose Melphalan and Autologous Stem Cell Transplantation With Implications for Adoptive T-Cell Therapy

**DOI:** 10.3389/fonc.2020.568056

**Published:** 2020-12-11

**Authors:** Thomas Mika, Swetlana Ladigan-Badura, Abdelouahid Maghnouj, Bakr Mustafa, Susanne Klein-Scory, Alexander Baraniskin, Sascha Döhring, Ilka Fuchs, Stephan Ehl, Stephan A. Hahn, Roland Schroers

**Affiliations:** ^1^ Department of Medicine, Hematology and Oncology, Ruhr-University Bochum, Bochum, Germany; ^2^ Department of Molecular Gastrointestinal Oncology, Ruhr-University Bochum, Bochum, Germany; ^3^ Center for Chronic Immunodeficiency, Faculty of Medicine, University of Freiburg, Freiburg im Breisgau, Germany

**Keywords:** gene expression, lentiviral transduction, lymphocyte proliferation, chimeric antigen receptor, melphalan, multiple myeloma, T-cell biology

## Abstract

In relapsed and refractory multiple myeloma (MM), adoptive cell therapies (ACT) including CAR-T-cells are under clinical investigation. However, relapse due to T-cell exhaustion or limited persistence is an obstacle. Before ACT are considered in MM, high-dose (HD) melphalan followed by autologous stem-cell transplantation (autoSCT) has been administered in most clinical situations. Yet, the impact of HD chemotherapy on T-cells in MM with respect to ACT is unclear. In this study, T-lymphocytes’ phenotypes, expansion properties, lentiviral transduction efficacy, and gene expression were examined with special respect to patients following HD melphalan. Significant impairment of T-cells’ expansion and transduction rates could be demonstrated. Expansion was diminished due to inherent disadvantages of the predominant T-cell phenotype but restored over time. The quantitative fraction of CD27^−^/CD28^−^ T-cells before expansion was predictive of T-cell yield. Following autoSCT, the transduction efficacy was reduced by disturbed lentiviral genome integration. Moreover, an unfavorable T-cell phenotype after expansion was demonstrated. In initial analyses of CD107a degranulation impaired T-cell cytotoxicity was detected in one patient following melphalan and autoSCT. The findings of our study have potential implications regarding the time point of leukapheresis for CAR-T-cell manufacturing. Our results point to a preferred interval of more than 3 months until patients should undergo cell separation for CAR-T therapy in the specific situation post-HD melphalan/autoSCT. Monitoring of CD27^−^/CD28^−^ T-cells, has the potential to influence clinical decision making before apheresis in MM.

## Introduction

In recent years, genetically engineered T-cells have revolutionized cancer therapy by the introduction of chimeric antigen receptors (CARs) into clinical practice. Stably introduced by viral transduction into T-cells, CARs allow effector cells to identify and kill cancer cells in a specific way. In diffuse large B-cell lymphoma (DLBCL) and acute B-cell leukemia (B-ALL), CAR-T therapy against CD19 has been implemented in treatment strategies for refractory and relapsed disease. Similarly, in multiple myeloma (MM) the efficacy of CAR-T cells is currently tested in advanced clinical trials (1–3). However, after impressing initial responses a majority of patients eventually relapse. In MM, T-cell inherent disadvantages, such as exhaustion and limited persistence, are believed to play a major role in relapse after CAR-T treatment (4).

The manufacturing process of CAR-T-cells is highly controlled and standardized to achieve high quality, treatment safety, and a low production failure rate. For appropriate expansion and transduction, T-cells need to be activated, commonly by CD3/CD28 stimulatory agents. Activation *via* both, CD3 and CD28, leads to rapid proliferation, while stimulation of CD3 without co-stimulation results in anergy. Moreover, the success of VSV-G based viral transduction in T-cells seems to rely on upregulation of LDL-Receptor and its family members, as they provide the docking receptor for viral entry into the cells (5).

Both, transduction efficacy and T-cell expansion capacity strikingly influence the quality of the final product, the latter with an independent influence on *in vivo* life span and engraftment (3). Additionally, the phenotype of the infused T-cells is relevant for treatment success. T-cell phenotypes can be characterized by the expression of multiple surface proteins, assigning them to naïve T-cells (T_N_; CCR7^+^,CD62L^+^, CD45RA^+^), central memory T-cells (T_CM_; CCR7^+^, CD62L^+^, CD45RA^−^), effector memory T-cells (T_EM_; CCR7^−^,CD62L^−^, CD45RA−), and T-effector cells (T_Eff;_ CCR7^−^,CD62L^−^, CD45RA^+^), respectively (6, 7). In the setting of CAR-T therapy, memory T-cells and T_Eff_ engraft and proliferate less as compared to T_N_ (8, 9). Moreover, presence of T_EM_ and T_CM_ in the apheresis product have been described to augment differentiation of T_N_ into T_EM_ and T_Eff,_ *via* Fas-ligand (Fas-L) interactions (10, 11).

Considering the impact of T-cell phenotypes on treatment success, the influence of chemotherapy on T-cells in patients undergoing CAR-T therapy is under investigation. In recent studies of DLBCL, senescent CD27^−^/CD28^−^-T-cells have been demonstrated to enrich in apheresis products after multiple cycles of chemotherapy (12). Besides, proliferation and differentiation of T-cells during expansion, as well as anti-tumor activity of CAR-T-cells after transfusion are impaired after chemotherapy (10, 12). In line with this data, recent clinical trials in MM emphasize improved anti-tumor activity of CAR-T-cells with less chemotherapy before apheresis (13).

For eligible patients with MM, induction therapy followed by high-dose chemotherapy and autologous stem-cell transplantation (autoSCT) represents a standard first-line therapy. The alkylating agent melphalan is the most commonly applied conditioning therapy prior to autoSCT in MM (14). Alkylating agents have a major impact on lymphocyte biology (15–17). Even after numeric reconstitution the function of T-lymphocytes remains impaired (16, 18). At this point, the impact of high-dose (HD) melphalan on T-cell biology is unsolved.

Currently, there are no in-depth studies of T-cell biology in relation to cytotoxic pharmacotherapy including HD regimens in the light of CAR-T treatments. Accordingly, recommendations at which time point lymphocyte apheresis result in optimal starting conditions for CAR-T-cell production. Hence, we examined the impact of HD melphalan on T-cell biology including subset distribution, expansion properties, lentiviral transduction efficacy, and global gene expression.

## Materials and Methods

### Patients and Healthy Donors

All patients were treated at the Universitätsklinikum Knappschaftskrankenhaus Bochum and had hematologic confirmed multiple myeloma (revised IMWG criteria 2014). The ethical committee of the Ruhr-University Bochum approved the study (No. 18-6462). Participants were HIV negative, gave their informed consent, and were recruited from three different groups: healthy donors, untreated MM, and MM 3 months after autoSCT. The groups were not matched for age or sex.

Healthy donors were without acute disease, and all participants were free of lymphocyte influencing medication (e.g., prednisolone). Prior to autoSCT all MM patients had received bortezomib, cyclophosphamide, and dexamethasone. Peripheral blood cells were collected at different time points. Cell counts and laboratory data were determined in parallel.

### T-Cell Expansion From Peripheral Blood Mononuclear Cells

**Figure 1:**
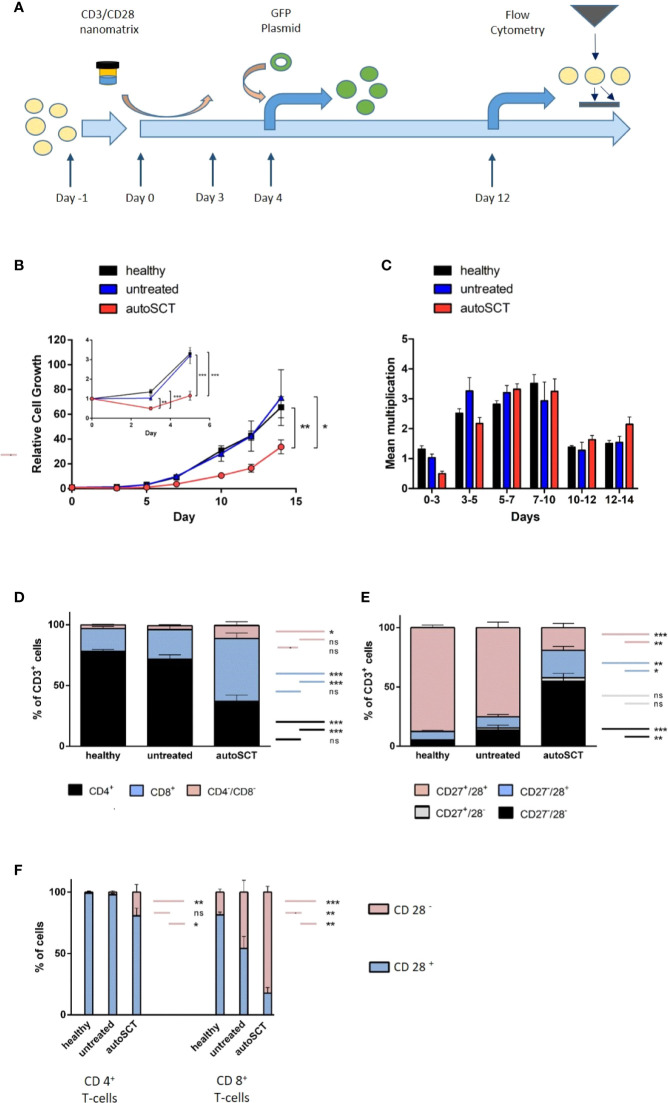
**(A)** T-cell expansion protocol. After an initial plastic adherence step (day -1), cells were activated by CD3/28-nanomatrix (day 0–3). From day 0 on, cells were cultivated in presence of IL-2. Transduction was performed on day 4, flow cytometry on day 12. **(B)** *Large:* relative cell growth of T-cells over 14 days (healthy n=10, untreated n=5, autoSCT n=9). After autoSCT, T-cells’ mean relative cell growth was significantly lower (day 14: autoSCT *vs.* healthy: p=0.007, autoSCT *vs.* untreated: p=0.047). *Small*: expansion in the first 5 days. Initial decrease of cells after autoSCT was significant (day 3: autoSCT *vs.* healthy p<0.001, autoSCT *vs.* untreated p=0.003; day 5: autoSCT *vs.* healthy: p<0.001, autoSCT *vs.* untreated: p<0.001). **(C)** Mean multiplication of cells in each measurement interval. The significant difference in speed of growth was only apparent in the first days. **(D)** Fraction of CD4^+^, CD8^+^, and CD4^−^/CD8^−^ double negative T-cells before expansion. Double negative cells possibly indicated extrathymic lymphopoiesis after autoSCT (16). **(E)** Fractions of CD27/CD28 expressing T-cells before expansion. CD27^−^/CD28^−^T-cells increased significantly after autoSCT (compared to healthy and untreated: both p<0.0001). **(F)** Expression of CD28 in CD4^+^ and CD8^+^ T-cells. *Red*: fraction of CD28^−^ T-cells, *blue*: fraction of CD28^+^ T-cells. No difference occurred between healthy individuals and untreated myeloma patients in CD4^+^ T-cells, whereas patients’ T-cells after autoSCT displayed lower CD28 expression (p=0.010 and p=0.039). In contrast, untreated patients showed lower CD28 expression in CD8^+^ T-cells compared to healthy individuals (p = 0.006). In patients after autoSCT, the differences between healthy individuals and untreated patients were more profound (p < 0.001 and p=0.002). (Symbols and bars: * = p < 0.05, ** = p < 0.001, *** = p < 0.0001; long colored bars: statistical comparison of fractions in column 1 *vs.* column 3, short colored bars: statistical comparison of fractions in column 2 *vs.* column 3 or column 1 *vs.* 2, respectively).

Peripheral blood mononuclear cells (PBMC) were collected using standard density centrifugation. After 24h plastic adherence, cells were counted and adjusted to 1–2 x 10^6^ cells/ml in complete medium [(Roswell Park Memorial Institute) RPMI + 10% FCS + 1% penicillin/streptomycin]. Cell activation was performed with CD3/CD28-nanomatrix (TransAct, Miltenyi Biotec™, Bergisch Gladbach, GER) according to manufacturer’s instructions in presence of interleukin 2 (IL-2). Cells were counted on days 0, 3, 5, 7, 10, 12, and 14 (**Figure 1A**). Cell counts and relative cell growth were calculated based on dilution and splitting of cells during the expansion period. For detailed description of the cell expansion protocol and expansion in presence of antibodies/conditioned medium please refer to the **Supplemental Materials**.

### Lentiviral Transduction of T-Cells

Green fluorescent protein (GFP) coding lentivector was produced using a three-plasmid system and CaCl_2_ transfection of HEK293T cells, as reported (19). Lentiviral supernatant was concentrated by centrifugation in Amicon^®^ Ultra-15 Centrifugal Filter Devices (3,500xg, 4°C, ≈ 15min.). The concentrated supernatant was stored at −80°C. Lentiviral transduction of T-cells was carried out on day 4 of T-cell expansion using lentiviral supernatant (supplemented with 4 ng/µl polybrene and 200 IU/ml IL-2; **Figure 1A**). The cell medium was changed after 24h (RPMI+IL-2). For uniform and comparable results, aliquots of the same lentiviral stock were used in all transduction experiments.

### Flow Cytometry and Cell Sorting

Flow cytometry (FCM) of PBMCs before expansion was performed with commercially available antibodies against CD3, CD4, CD8, CD27, CD28, CD45 (Beckman Coulter™, Krefeld, GER) at day 0. The gating strategy excluded other blood cells than CD3+ lymphocytes, and the lentiviral transduction efficacy 72h after transduction (day 7 after beginning of expansion) was determined by quantitation of GFP-positive T-cells by FCM.

T-cell differentiation during cell expansion was assessed at day 12 (**Figure 1A**). Antibodies against CD3, CD45RA, CD62L, CCR7, CD27, CD28 (all Miltenyi Biotec™), and CD4 (Santa Cruz™, Dallas, Tx, US) were applied. Low-density-lipoprotein receptor (LDL-R) expression on CD3+ T-cell surfaces was determined with an allophycocyanin (APC)-labeled anti-LDL-R antibody (R&D™, Minneapolis, MN, US). The mean of APC-signal was used for further analyses. Flow-cytometry data were analyzed with FlowJo v10.6.1. Sorting of CD3^+^ cells for the purpose of gene expression analysis was carried out on a MoFlo Astrios EQ apparatus (Beckman Coulter™). The gating strategy excluded other than T-cells based on CD3-staining.

### Gene Expression Analysis and Data Processing

An amount of 100 ng of total RNA was hybridized to Agilent whole-genome expression microarrays (Human GE 4x44K, v2 G4845A, AMADID 026652, Agilent Technologies™, Santa Clara, CA, US). RNA labeling, hybridization, and washings were carried out according to manufacturer’s instructions. Images of hybridized microarrays were acquired with a DNA microarray scanner (Agilent G2505B) and features were extracted using the Agilent Feature Extraction image analysis software (AFE) version A.10.7.3.1 with default protocols and settings. The AFE algorithm generates a single intensity measure for each feature, referred to as the total gene signal (TGS), which was used for further data analyses using the GeneSpring GX software package version 14.9.1. AFE-TGS were normalized by the quantile method. Subsequently, data were filtered on normalized expression values.

For identification of differentially expressed genes, only entities with at least two out of the total number of samples and values within the selected cut-off (50^th^ percentile) were further included in the data analysis process. Using the GeneSpring GX software package version 14.9.1, differentially expressed genes were identified *via* moderated t-test. Finally, only messenger RNAs (mRNAs) with a ≥ 2fold change in the microarray analyses were further considered. Gene set enrichment analysis (V 4.0.3.) software was used for analysis of predefined gene sets.

### T-Cell Degranulation Assay

Degranulation of CD8^+^ T-cells was assessed in the laboratories of the Advanced Diagnostics Unit at the CCI at the University Freiburg. Assays were carried out as previously reported (20).

### Digital-Droplet PCR

Digital-Droplet PCR (ddPCR) was used for determination of mean GFP-copies per cell in genomic DNA of GFP-transduced T-cells. Workflow was performed as previously published (21). In brief, PCR-mix (Primer/Probe-Mix, SuperMix, EcoRI, DNA) was incubated for 10 min at 37°C followed by the PCR reaction: 95°C 10 min, 40 cycles of 94°C 30 s, and 60°C 30 s, followed by 98°C 10 min. The readout was performed on the QX200 droplet reader (BioRad™, Feldkirchen, GER). The GFP-Probe was FAM labeled. Hex-labeled RPPH1 was used as housekeeper. Mean GFP copy numbers were calculated by division of GFP and RPPH1 signals.

### Statistical Analysis

All statistical analyses and data plots were carried out with GraphPad Prism software (version 5). If not otherwise specified, error bars represent standard error of mean (SEM). Regression analysis, Pearson correlation (r^2^), and two-tailed t-test were used for correlation analyses. Symbols: * = p<0.05, ** = p<0.001, *** = p<0.0001.

## Results

### Patients and Samples

We isolated PBMCs from 27 individuals and assessed pre-expansion T-cell phenotype as described above (n=10 healthy, n=7 untreated, and n=10 individuals after autoSCT). In T-cells collected from 24 participants, expansions with CD3/28-nanomatrix and IL-2 were performed (n=10 healthy, n=5 untreated, and n=9 individuals after autoSCT). Indeed, not all participants’ T-cells were expandable due to limited cell counts after cells were used for further gene expression and flow cytometry analyses (n=3 were excluded). Overall, samples from 20 individuals could be transduced with GFP (n=6 healthy, n=5 untreated, and n=9 after autoSCT). Patient demographics and basic laboratory parameters are depicted in **Table 1**.

**Table 1:** Baseline characteristics of untreated patients and patients after autoSCT (phenotype and expansion analyses).

Patient No.	Group	Age	WBC (10^3^/µl)	ALC (10^3^/µl)	Platelets (10^3^/µl)	ß2-M (mg/l)
**3**	untreated	65-69	7.600	2.560	241.0	3.40
**4**	untreated	80-84	5.300	0.340	199.0	4.70
**7**	autoSCT	65-69	7.300	2.670	138.0	5.90
**8**	autoSCT	60-64	7.500	3.110	215.0	6.33
**9**	autoSCT	55-59	7.200	0.940	303.0	1.69
**14**	autoSCT	55-59	5.200	2.100	283.0	2.43
**16**	autoSCT	55-59	6.700	0.450	318.0	6.14
**18**	untreated	60-64	3.600	1.120	271.0	2.04
**19**	untreated	55-59	3.900	0.980	164.0	1.97
**20**	untreated	50-54	8.200	2.640	283.0	2.12
**21**	autoSCT	70-74	3.600	0.770	129.0	3.81
**24**	autoSCT	60-64	3.600	0.840	168.0	3.08
**27**	autoSCT	65-69	3.800	0.860	231.0	1.80
**28**	autoSCT	75-80	2.400	0.600	189.0	1.98
**29**	autoSCT	65-69	1.900	0.360	170.0	2.11
**30**	untreated	55-59	10.100	1.360	289.0	2.12
**31**	untreated	60-64	3.500	1.620	98.0	1.90

WBC, white blood cell count; ALC, absolute lymphocyte count; B2M, β2-microglobulin.

### T-Cell Expansion and T-Cell Homeostasis Following High-Dose Melphalan and AutoSCT

To evaluate the impact of HD melphalan and autoSCT on T-cell expansion we compared T-cells collected from healthy donors, untreated patients, and patients 3 months after melphalan/autoSCT, respectively. Relative lymphocyte growth was significantly diminished after autoSCT as compared to both, healthy donors and untreated patients. Mean of relative cell growth on day 14 was 33.77 (SEM ±5.60) after autoSCT, compared to 65.7 (± 8.55) in healthy individuals (p=0.007). Mean relative cell growth of untreated patients was 73.42 (± 22.25), which was not different (p=0.65) in healthy donors at the same time, but significantly higher compared to patients after autoSCT (p=0.047; **Figure 1B**, large). Analysis between each measuring point revealed an initial decrease of T-cells between days 0 and 3 in all patients after autoSCT. Overall, cell counts did not increase for the first 5 days in these patients. On the contrary, cells collected from healthy donors demonstrated immediate growth (**Figure 1B**, small; **Figure 1C**). Differences in relative cell growth in patients post-autoSCT were significant at days 3 and 5 compared to healthy donors (*day 3*: autoSCT = 0.50 (± 0.08), healthy donors = 1.32 (± 0.11), p<0.001; *day 5*: autoSCT = 1.16 (± 0.23), healthy donors = 3.21(± 0.17), p<0.001). Compared to untreated patients, cell growth at days 3 and 5 differed as well to a significant extent (*day 3:* untreated = 1.03, p=0.003; *day 5:* untreated = 3.20, p<0.001). After 5 days, the dynamic of proliferation was roughly the same in all groups (**Figure 1C**). These results demonstrated that differences in cell growth were not due to myeloma per se, but T-cellular expansion capacity was impaired subsequent to autoSCT.

To gain further insight into T-cell characteristics at the beginning of the expansion, we analyzed T-cell phenotypes by flow cytometry prior to expansion. Significant differences in the composition of PBMCs 3 months after HD melphalan compared to both, healthy donors and untreated patients, could be demonstrated. The CD3^+^ cell fraction in the buffy coat was lower in peripheral bloods after autoSCT (**Supplementary Figure S1**). However, the amount of CD3^+^ in the PBMCs did not correlate with overall cell growth (R^2 =^ 0.13; **Supplementary Figure S2**). The CD4:CD8 ratio, which is tightly regulated in healthy donors, was significantly altered following melphalan/autoSCT. T-cells’ phenotype shifted toward CD8^+^ with CD4:CD8 ratios of 3.92 in the healthy population as compared to 0.7 after melphalan/autoSCT (p<0.001; **Supplementary Figure 1D**). Additionally, we found an increased fraction of CD4^−^/CD8^−^ T-cells subsequent to melphalan/autoSCT compared to healthy T-cells (p=0.02). In untreated myeloma patients, CD4:CD8 ratios were significantly decreased to 2.54 as compared to healthy donors (p=0.030), however, the ratios were still positive and significantly higher as compared to patients after autoSCT (p=0.023).

Analysis of CD27 and CD28 co-expression revealed a significant decrease of CD27^+^/CD28^+^ T-cells and a correlated relative increase of CD27^−^/CD28^−^ T-cells after autoSCT (**Figure 1E**). In healthy individuals, mean fractions of CD27^+^/CD28^+^ T-cells and CD27^−^/CD28^−^ T-cells in the buffy coat were 87.5% (± 1.83) and 4.2% (± 1.34), respectively. On the opposite, the mean fraction of CD27^+^/CD28^+^ T-cells and CD27^−^/CD28^−^ T-cells after autoSCT were 19.9% (± 3.91%) and 55.3% (± 7.24%), respectively (both p<0.001). In untreated myeloma patients, a slight increase of CD27^−^/28^−^ T-cells to a mean fraction of 13.6% (± 4.13) and decrease of CD27^+^/CD28^+^ T-cells to a mean fraction of 75.2% (± 4.53) in comparison to healthy donors could be demonstrated (p=0.02 and p=0.013, respectively). Still, both fractions were significantly different from patients after autoSCT (both p<0.001). In addition, we found more CD27^−^/CD28^+^ T-cells after autoSCT as compared to healthy donors and untreated patients (p=0.003 and p=0.007, respectively; **Figure 1E**).

Next, we analyzed if the changes in CD28 expression were uniform for both, CD4^+^ and CD8^+^ T-cells. Following autoSCT a reduction of CD28 expression in both T-cell subtypes could be demonstrated (**Figure 1F**). Remarkably, this decrease in CD28 expression was much more pronounced in CD8^+^ T-cells as compared to CD4+ T-cells. Also, CD28 co-expression in T-cells collected from untreated myeloma patients was shown lower in comparison to T-lymphocytes from healthy individuals (**Figure 1F**, right).

### CD27^−^/CD28^−^ T-Cells and T-Cell Expansion Following High-Dose Melphalan/AutoSCT

**Figure 2:**
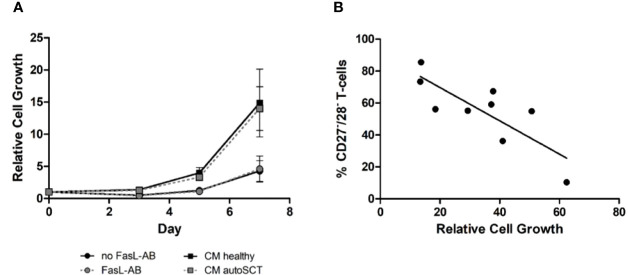
**(A)** Expansion of T-cells after autoSCT in presence of Fas-L antibody (dots), and expansion of healthy individuals’ T-cells in conditioned medium (squares). No difference was seen in expansion properties, indicating T-cell inherent disadvantages after autoSCT. For functional testing of the Fas-L antibody please refer to the supplemental material (**Supplementary Figure S5**). **(B)** Correlation of CD27^−^/CD28^−^ T-cells in the buffy coat at day 0 and relative expansion at day 14 of T-cells after autoSCT. Expression of CD27 and CD28 could predict expansion properties 3 months after autoSCT (R^2^ = 0.65).

To exclude Fas-Ligand (Fas-L) mediated fratricide, we expanded lymphocytes collected from patients after HD melphalan and autoSCT in the presence of a Fas-L antibody **(** **Figure 2A** **, dots)**. In order to exclude growth inhibiting effects of soluble factors, released by cells after autoSCT, we expanded cells of healthy donors in conditioned medium (CM) derived either from healthy donors or patients after melphalan/autoSCT (**Figure 2A** **, squares**). No differences in cell growth where observed, when T-cells were cultured in CM. Furthermore, addition of Fas-L-antibody did not alter relative cell expansion.

In summary, we concluded that intrinsic disadvantages of patients’ T-cells after melphalan/autoSCT caused the diminished expansion. Interestingly, we found a correlation between the fraction of CD27^−^/CD28^−^ T-cells in the buffy coats and the relative cell growth during expansion (**Figure 2B** **;** R^2^= 0.65).

### Analysis of T-Cell Regeneration

**Figure 3:**
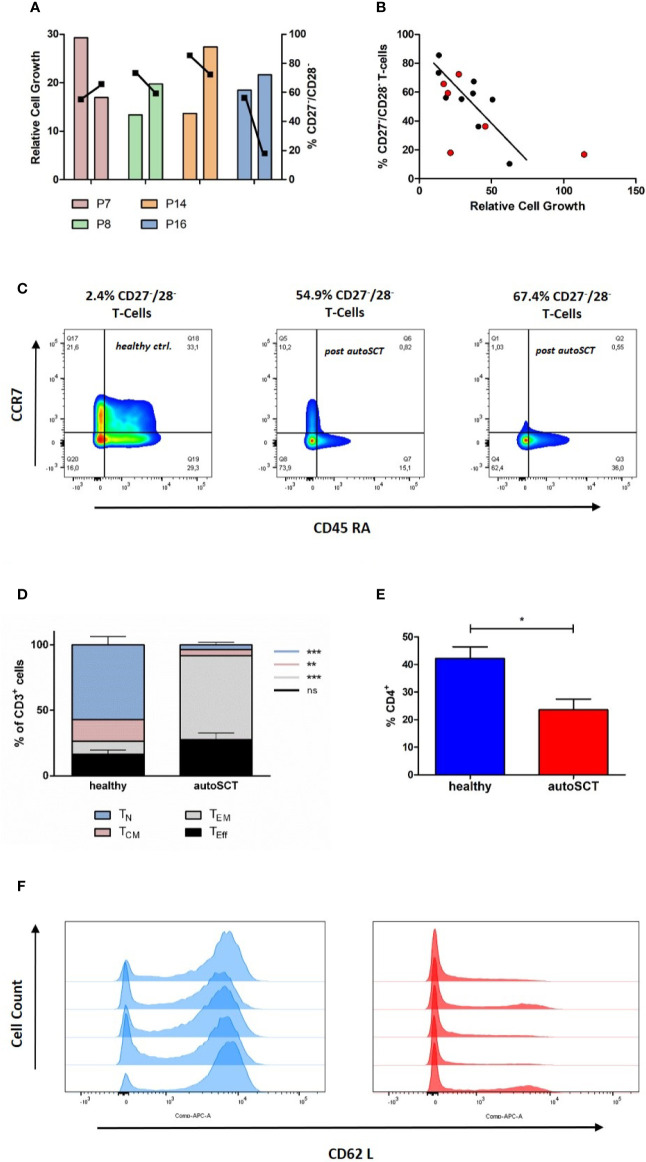
**(A)** Relative cell growth and CD27/CD28 expression at two different time points (*left bar*: 3 months after autoSCT, *right bar:* follow-up). When CD27^−^/CD28^−^ T-cells decreased over time, relative expansion increased (bars: relative cell growth, squares: % of CD27^−^/CD28^−^ T-cells). Further information in **Supplementary Table S1**. **(B)** Correlation of CD27/CD28 expression and relative expansion. [black: 3 months after autoSCT (as in **Figure 1G**), red: six additional patients]. **(C)** Representative flow cytometry of CCR7 and CD45RA expressing T-cells (CD3^+^) on day 12. The fraction of CD27^−^/CD28^−^ T-cells at the beginning of expansion is indicated. *Left:* healthy individual; *middle and right:* autoSCT. **(D)** T-cell phenotype at day 12 based on CCR7 and CD45RA expression (T_N_ p< 0.001, T_CM_ p=0.0012, T_EM_ p<0.001, T_Eff_ ns; each group n=5). **(E)** Fraction CD4^+^positive T-cells after expansion (each group n=5). **(F)** Histogram of CD62L expression at day 12. *Blue:* healthy individuals, *red*: patients after autoSCT.

Next, we sought to investigate if the T-cell expansion capacity recovers over time. Therefore, blood samples from four myeloma patients were collected 7–10 months following HD melphalan and autoSCT. In three of four patients the fraction of CD27^−^/28^−^ T-cells decreased as compared to the initial measurements (**Figure 3A**). As expected, in patients with decreased CD27^−^/CD28^−^ T-cells the cellular expansion capacity increased in comparison to the first measurement (**Figure 3A**). This initial data indicated that the expansion capacity can in principle restore over time. However, the process appeared not uniform, and some patients may not recover. Thus, we believe that it is important to obtain reliable information to the cellular expansion capacity in the context of adoptive T-cell therapy. Accordingly, we tested if CD27^−^/CD28^−^ T-cell fraction is able to predict expansion, regardless of the time passed since autoSCT. We collected two additional PBMC samples 7 and 13 months post-autoSCT. Subsequently, we compared the expansion capacities to the amount of CD27^−^/CD28^−^ T-cells in overall six samples obtained beyond 3 months after autoSCT. In **Figure 3B** the results are summarized in combination with the regression curve of previous samples 3 months after autoSCT. Overall, a significant correlation between CD27^−^/CD28^−^ fraction and the expansion capacity (R^2 =^ 0.65, p=0.009) was observed.

T-cells collected from one patient expanded worse than expected from the correlation curve (CD27^−^/CD28^−^: 18%, relative cell growth: 21.6). We found this patient having 40% of all T-cells in the buffy coat to be CD4^−^/CD8^−^. Origin of double-negative T-cells after autoSCT is not fully understood (16). However, this may explain worse expansion as these T-cells have immunosuppressive function in case of double-negative regulatory T-cells and need special culture conditions to expand in case of *γ*/δ T-cells (22–24).

### T-Cells of Patients After AutoSCT Show Effector-Memory and Effector Phenotype After *In-Vitro* Expansion

To unravel the impact of autoSCT not only on T-cell phenotypes before, but also after *in vitro* expansion, we investigated the T-cell differentiation after 12 days of expansion in a subset of participants from both groups, 3 months after autoSCT and healthy donors (each n=5), respectively. In comparison to healthy individuals, patients post-autoSCT showed higher fractions of T_Eff_ and T_EM_ subsets, as characterized by CD45RA and CCR7 expression (**Figures 3C, D** **)**. Besides, we found lower fraction of CD4^+^ T-cells following melphalan/autoSCT and the expression of the homing marker CD62L was significantly reduced (**Figures 3E, F** **)**.

In a small subset of untreated myeloma patients (n=2) we found significantly more effector, but also less T_EM_ compared to patients after autoSCT (T_Eff_ p=0.004 and T_EM_ p=0.010; **Supplementary Figure S3**). These results indicated myeloma inherent effects, resulting in decreased amounts of naïve T-cells after expansion. However, we are aware that this finding has to be confirmed in a larger cohort.

### Lentiviral Transduction Efficacy Following High-Dose Melphalan and AutoSCT

**Figure 4:**
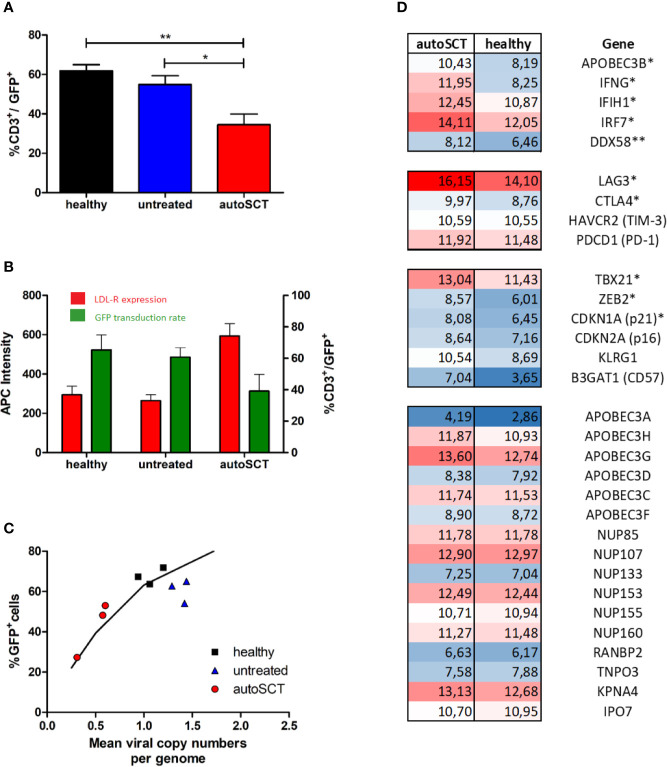
**(A)** Mean transduction efficacy (± SEM) assessed by flow cytometry 3 days after transduction with GFP-lentivirus (healthy n=6, untreated n=5, autoSCT n=9). **(B)** Expression of LDL-R (red columns, mean intensity ± SEM) at the time of transduction with GFP-lentivirus and subsequent transduction efficacy (green columns, mean ± SEM) (healthy n=4, untreated n=3, autoSCT n=2). No correlation between expression of LDL-R at the cell surface and number of transduced cells was observed. **(C)** Correlation of GFP^+^ T-cell fraction and mean viral copy numbers per genome assessed by ddPCR. The black line indicates the expected fraction of GFP^+^ T-cells based on Poisson’s statistic (25). Results matched expected values based on Poisson’s statistic. **(D)** RNA-array analysis of sorted T-cells (CD3^+^). Numbers indicate normalized signal intensity (log2 scale) as described in supplement. *Top:* significantly upregulated transcription of antiviral genes. *Middle:* transcription of genes involved in exhaustion and senescence. *Bottom:* non-significantly regulated genes. A trend to higher transcription of APOBEC3G and APOBEC3H in T-cells after autoSCT was detected (* = p < 0.05, ** = p < 0.01).

In a next step, we assessed the lentiviral transduction efficacy in T-cells by flow-cytometry following lentiviral transduction with GFP. Following melphalan/autoSCT the transduction efficacy was significantly diminished. Here, the mean fraction of GFP-expressing T-cells was 33.4% (± 4.87%). On the contrary, in healthy donors and untreated myeloma patients, the means of GFP+ T-cells were 61.8% (± 3.14%) and 54.90 (± 4.48%), respectively (p=0.002 and p=0.02; **Figure 4A**).

Accordingly, we examined the mechanisms for the impaired transduction efficacy by VSV-G pseudo-typed lentivectors. First, we investigated the lentiviral entry into T-cell. As previously reported (5, 26), upon activation by CD3/CD28 stimulation T-lymphocytes become susceptible to viral transduction by upregulation of LDL-R and it’s family members, which represent the main entry for VSV-G based viral transduction. In our experiments, a dynamic expression of LDL-R during T-cell activation could be demonstrated in flow cytometry. We found an upregulation within 4 days after stimulation, followed by LDL-R downregulation during further expansion (**Supplementary Figure S4**). However, the LDL-R expression was not impaired in patients subsequent to melphalan/autoSCT, and, no correlation with transduction efficacies could be demonstrated (**Figure 4B**).

### Reduced Transduction Efficacy Is Caused by Disturbed Lentiviral Genome Integration

Next, we examined the integration of lentiviral complementary DNA (cDNA) into the host genome. We performed ddPCR on extracted genomic DNA of GFP-transduced T-cells in three samples of each group. In general, viral transduction follows Poisson’s statistics (25). Based on Poisson’s formula, the percentage of GFP-expressing cells can be calculated by the mean viral copy number in a cell suspension and vice versa. If decreased integration of viral cDNA into T-cell genomes were responsible for decreased transduction efficacy, Poisson’s formula would still hold. If not, altered transcription or translation of the transduced gene could be accountable. Thus, we correlated the mean viral copy number with the percentage of GFP expressing cells and found good correlation, indicating reduced integration of the transduced gene (**Figure 4C**).

Different proteins have the potential to reduce viral integration into the host’s genome by interfering with cDNA-synthesis and nuclear transport (27). Thus, we sought for differentially expressed genes involved in the cells’ antiviral defense. We performed mRNA-array analysis on four samples at the time of transduction, two derived from healthy individuals and two from patients after autoSCT (**Figure 4D**). All samples were sorted for CD3^+^ cells by flow-cytometry, to exclusively analyze T-cells. From all genes analyzed, we looked for differential expression of genes known to be important for antiviral activity during viral replication and integration (27–30). Additionally, we performed gene-set enrichment analysis (GSEA) with a predefined gene set of interferon-controlled antiviral genes.

**Figure 5:**
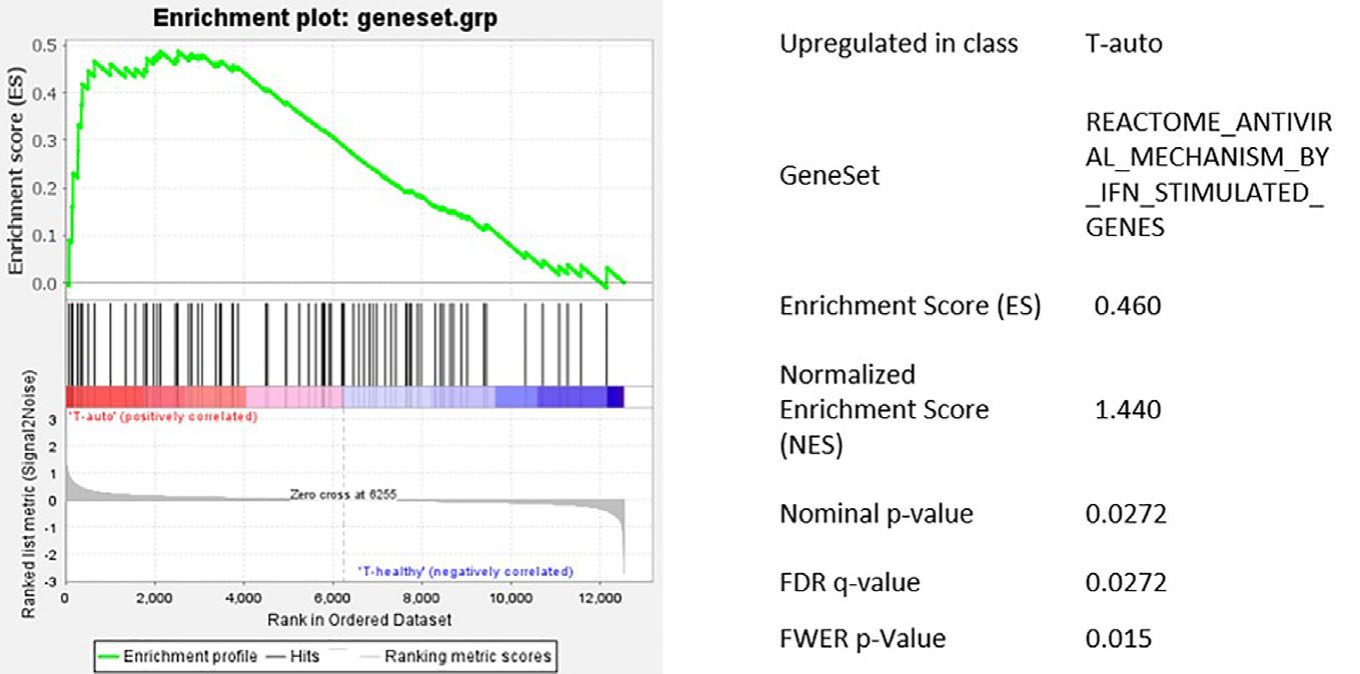
Gene set enrichment analysis of the predefined gene set for IFN regulated antiviral genes. Antiviral genes were significantly upregulated in T-cells after HD melphalan and autoSCT.

We found a significantly higher transcription of Interferon-gamma (*IFNG*) in T-cells after melphalan/autoSCT. Also, genes encoding for antiviral proteins like APOBEC3B, DDX58, IRF7, IFIH1 were significantly upregulated. In line with our flow-cytometry findings, we found increased transcription of T-cell exhaustion and senescence genes (*CTLA4, LAG3, ZEB2, TBX21, p21*) following HD melphalan and autoSCT. Proteins involved in nuclear transport of viral DNA were not altered, making an influence on viral integration unlikely. Finally, GSEA with the predefined gene set showed significant upregulation of interferon stimulated antiviral genes (**Figures 4D** and **5**).

### Impaired T-Cell Cytotoxicity Following High-Dose Melphalan and AutoSCT

**Figure 6:**
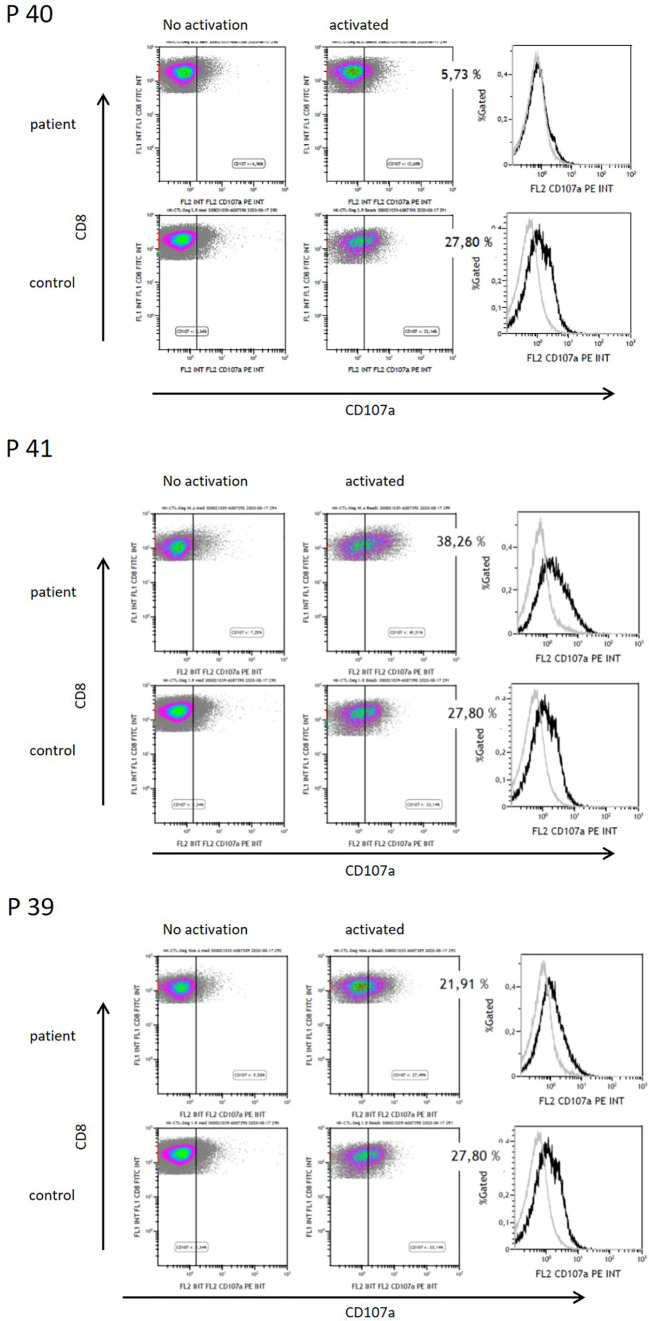
Functional analysis of CD8^+^ T-cells by CD107a degranulation of three patients after HD melphalan and autoSCT. Patient 40 showed decreased CD107a degranulation, whereas patients 41 and 39 had normal functionality.

As direct cytotoxic function of CD8^+^ T-cells is critical for the success of adoptive T-cell therapy, we investigated T-cells after autoSCT in preliminary determination of cytotoxic degranulation. We analyzed CD107a degranulation in CD8^+^ T-cells collected from three patients after autoSCT. In one of three patients, impaired degranulation was observed as compared to unimpaired CD8^+^ T-cells’ degranulation in blood samples collected from healthy donors (**Figure 6**).

## Discussion

The present study focuses on the impact of HD chemotherapy and autoSCT on MM patients’ T-cells and their utility for adoptive cell therapies (ACT). Due to the advent of CAR-T therapy in MM a detailed knowledge of T-cells’ behavior and biology after first-line treatment in MM is of increasing interest, since most patients receive HD melphalan followed by autoSCT before CAR-T-cell manufacturing. We demonstrated that expansion and transduction of T-cells is significantly diminished 3 months after autoSCT. Taken together, both effects sum up and reduce the expected yields of T-cells after expansion. The probability of manufacturing failures is increased due to limited expansion, as the needed minimum cell count for efficient doses may not be achieved. Additionally, our data show that HD melphalan and autoSCT considerably influence T-cell phenotype before and after expansion with accumulation of exhausted and senescent T-cells and a consecutive loss of naive T-cells. Furthermore, in initial results of CD107a degranulation we present results of decreased cytotoxic function in CD8^+^ T-cells subsequent to HD melphalan and autoSCT. However, this data requires further confirmation and extension to impaired cytotoxic activity against specific target cells, e.g., impaired killing of B-cell maturation antigen (BCMA) expressing myeloma cells by antiBCMA-specific CAR-T cells.

Our findings are in line with other studies focusing on T-cell subsets in hematologic malignancies other than MM and emphasize the effect of chemotherapy on T-cells suitability for ACT in general (12, 18).

The influence of HD chemotherapy and autoSCT on T-cell phenotypes has been reported to be long-lasting (16, 17). However, some patients quickly progress after autoSCT and require early retreatments. Currently, the minimum time span applicable for T-cell apheresis following autoSCT to T-cell apheresis for ACT is not known. Here, we have tested patients 3 months after autoSCT. We observed the fraction of CD27^−^/CD28^−^ T-cells in the buffy coats (equaling the apheresis product) to correlate with the cell growth of T-cells during *in-vitro* expansion. Therefore, CD27^−^/CD28^−^ analysis before apheresis appears a helpful tool to predict expansion. Clearly, a meaningful threshold for T-cell manufacturing has to be further investigated in a larger cohort. Loss of CD27 after autoSCT was an intriguing finding of our study, which is known to impair T-cell function in adoptive cell transfer (31). Besides, CD28 expression was impaired substantially in both, CD4^+^ and CD8^+^ cells after HD melphalan and autoSCT. In untreated myeloma patients, CD28 expression was significantly altered compared to healthy individuals, but only in CD8^+^ T-cells, which is a known phenomenon in myeloma patients (32, 33). However, in our study these findings did not influence T-cell expansion. Considering durable remissions following HD melphalan in multiple myeloma recovery of T-cell immunity is an important question. The early results of this study with limited patient numbers and short follow-up times give an idea regarding post-melphalan restoration of proliferation capacity. However, in future studies analyzing long-term features of T-cell and also NK-cell biology post-cytotoxic treatments are required to optimize adoptive cell therapies with CARs.

Correlation of high amounts of CD27^−^/CD28^−^ T-cells in the apheresis product with lower *in vitro* expansion and anti-tumor activity has been recently shown in DLBCL patients (12, 34). In our study, we found higher accumulation of CD27^−^/CD28^−^ subsequent to autoSCT, compared to DLBCL patients in the latter studies. This was expectable considering the differences in treatment strategies and the lymphodepleting intensity of HD melphalan. However, these previous studies underline that the findings in our study result from intensive lymphotoxic melphalan and prolonged T-cell disturbance after autoSCT, as chemotherapy *per se* does not lead to a distinct T-cell damage (12, 16, 17).

Sufficient *in-vitro* expansion correlated with overall response rates (ORR) in several CAR-T studies (3, 9). The reduced expansion in our study appeared to be mainly driven by the augmentation of senescent and exhausted T-cells, which are known to proliferate worse as compared to naive T-cells (35).

Another finding of our study is the unfavorable phenotype of T-cells after expansion. In many trials investigating ACT and CAR-T-cell therapy, a higher fraction of T_CM_ and T_N_ in transferred lymphocytes, resulted in improved ORR and survival rates (3, 9, 10, 12, 36, 37). Thus, the shift toward T_EM_ and T_Eff_ subsequent to autoSCT demonstrated in our study and the loss of T_N_ will likely limit anti-tumor activity and persistence of T-cells and may cause relapses after CAR-T therapy in a MM patients.

**Figure 7:**
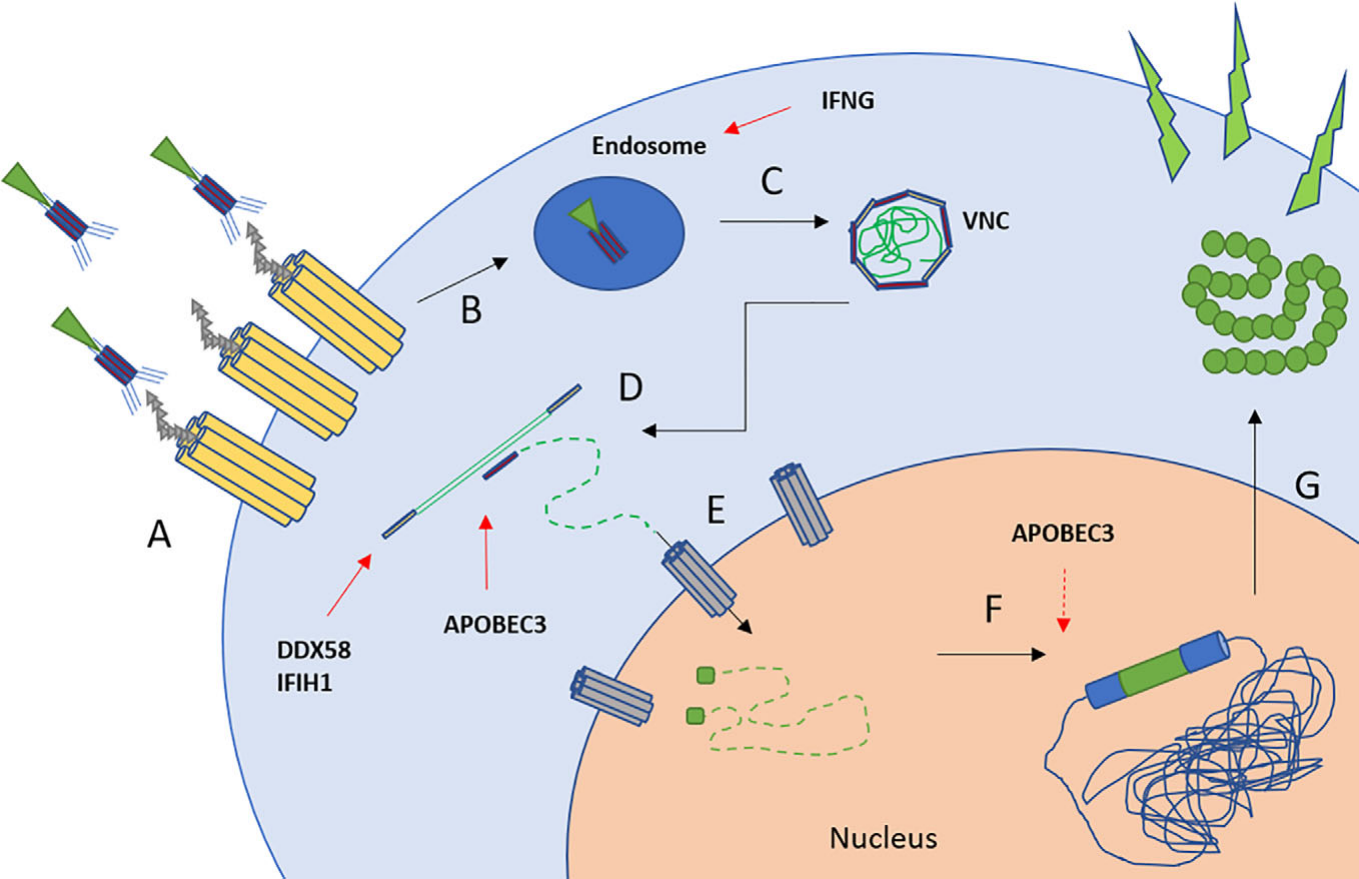
Scheme of lentiviral transduction and upregulated antiviral mechanisms (26, 27). **(A)** Binding of the viral particle to LDL-receptor and family members. **(B, C)** Uptake into endosomes and pH-dependent release of the viral nucleoprotein complex (VNC). IFNG inhibits endosome-cytoplasm transition (38). **(D)** Reverse transcription and cDNA synthesis. DDX58 and IFIH1 recognize RNA-strands followed by downstream activation (39, 40). APOBEC family members insert mutations into pro-viral DNA (41). **(E)** Transport of the cDNA into the nucleus by nuclear transporters, e.g., *via* NUP-family. **(F)** Integration into the host’s genome. APOBEC family members may interfere with LTR-sites. **(G)** Transcription and translation of the transferred gene.

Both, expansion capacity and transduction efficacy *via* a VSV-G based lentiviral system, were significantly impaired after autoSCT in our study. By using the same viral supernatant for each transduction and confirming the presence of viral docking receptors following autoSCT, chances of viral entry were equal in healthy and treated individuals. Moreover, mean viral copy numbers per cell (representing integrated viral genomes) and the fraction of GFP^+^ cells in flow cytometry (representing expression of the transferred gene) were as expected by Poisson’s statistics (25). Thus, we were able to exclude inferior transcription or translation to be the reason for limited transduction efficacy. The lower transduction efficacy after autoSCT resulted from less viral DNA being integrated into T-cells’ genomes. Thus, lower transduction efficacy is possibly the result of cytoplasmatic or nuclear defense mechanisms, preventing viral DNA to be integrated into the host’s genome. As a preliminary finding, GSEA confirmed upregulation of antiviral defense mechanisms in four individuals in our study, mainly induced by upregulation of IFN-related genes. **Figure 7** gives a graphic scheme of the antiviral mechanisms suggested in our experiments. Since the T-cell pool after autoSCT restores from pre-existing clones (16, 17), CD8^+^ T-cells are likely activated and capable of interferon-gamma production (42). This is underlined by the phenotypes found in our study after autoSCT, since T_EM_/T_CM_ and T_Eff_ are capable to release high amounts of IFN-gamma (42, 43). However, we are aware that the latter findings have to be confirmed in a larger cohort of patients and in preclinical tests, proving the mechanisms by avoiding antiviral mechanisms—e.g., with non-viral transduction.

The impact of autoSCT on T-cell subsets declines over time (16, 17), as most patients included in two clinical trials of a BCMA-CAR had undergone autoSCT before apheresis, and responded to CAR-T therapy anyway (2, 3). However, the interval from autoSCT to apheresis was not specified in the studies and patients relapsed due to limited T-cell persistence. Accordingly, it remains unclear if a longer time span from autoSCT to apheresis may improve ORR. Besides, the influence of immunomodulatory drugs, such as lenalidomide and pomalidomide, and anti-CD38 antibodies (daratumumab and isatuximab) on T-cell phenotypes remains unclear.

Our study provides first data suggesting restoration of expansion capacity over time and suggesting a preferred interval of more than 3 months until patients after autoSCT should undergo apheresis for CAR-T therapy. Furthermore, the fraction of CD27^−^/CD28^−^ T-cells may help to identify patients with unfavorable T-cells for ACT. Our findings highlight the differences of myeloma patients’ T-cells compared to T-cells of healthy donors when used for ACT, which should be considered in future research and clinical approaches. Non-viral transduction, e.g., by sleeping beauty transposition system (44), could overcome the antiviral mechanisms and enhance transduction efficacy.

## Data Availability Statement

The datasets presented in this study can be found in online repositories. The data discussed in this publication have been deposited in NCBI's Gene Expression Omnibus and are accessible via GEO Series accession number GSE148770 (https://www.ncbi.nlm.nih.gov/geo/query/acc.cgi?acc=GSE148770).

## Ethics Statement

The studies involving human participants were reviewed and approved by ethical committee of the Ruhr-University Bochum. The patients/participants provided their written informed consent to participate in this study.

## Author Contributions

TM, SL-B, AM, BM, SD, IF, and SK-S collected patient samples and performed the experiments.TM, AM, SH, SK-S, AB, SE, and RS designed the study and analyzed the data. TM, SH, and RS wrote the manuscript. All authors contributed to the article and approved the submitted version.

## Funding

The study was supported by a FoRUM grant (Ruhr-University Bochum, no.: F978-2020).

## Conflict of Interest

The authors declare that the research was conducted in the absence of any commercial or financial relationships that could be construed as a potential conflict of interest.
