# Supplementary material for: Altered T-Lymphocyte Biology Following High-Dose Melphalan and Autologous Stem Cell Transplantation With Implications for Adoptive T-Cell Therapy
Source: Front Oncol. 2020 Dec 11;10:568056. doi: 10.3389/fonc.2020.568056 (PMC7759611; doi:10.3389/fonc.2020.568056)
Supplement: Supplementary file 1 [file DataSheet_1.docx]

**Supplement**

**Material and Methods**

***Patients and sample preparation of Peripheral blood mononuclear cells (PBMC)***

All participants were HIV negative. Healthy donors were without acute diseases, and all patients had to be free of lymphocyte influencing medication (e.g. prednisolone). Prior to autoSCT all MM patients had received bortezomib, cyclophosphamide, and dexamethasone. Peripheral blood cells were collected at different time points. Cell counts and laboratory data were determined in parallel.

Peripheral blood mononuclear cells (PBMC) were collected using standard density centrifugation. Fresh whole-blood samples were collected in heparin tubes and further processed immediately by density gradient centrifugation with Lymphoprep^TM^ (800xg, 30 min, no brake). The PBMC layer was collected and washed twice with phosphate buffered saline (PBS). Cells were incubated in RPMI Medium containing 10% FCS and 1% Penicillin/Streptomycin (complete medium) overnight. Next day, non-adherent cells were collected by gently pipetting up- and down. The collected cell suspension was centrifuged, washed, and resuspended in complete medium (RPMI + 10% FCS + 1% Penicillin/ Streptomycin) in a concentration of 1-2 x10^6^ cells/ml. Subsequently, cells were phenotyped by flow cytometry and expanded as described below.

***T-cell expansion***

Cells were counted and diluted to 1-2 x 10^6^ cells/ml in complete RPMI Medium. They were incubated (5% CO_2_, 37°C) with 200 IU IL-2/ml (Novartis^TM^) and 10 µl human T-cell TransAct^TM^ /ml (Miltenyi Biotec^TM^). Cells were washed twice after 3 days and kept at a density of 1-2x10^6^ cells/ml. Cells were splitted as necessary every 2-3 days in fresh complete medium and IL-2. Cells were counted manually on days 0, 3, 5, 7, 10, 12 and 14. Cell counts and relative cell growth were calculated based on dilution and splitting of cells during expansion.

*Relative cell growth*: absolute cell count day n / absolute cell count day 0

*e.g.: Absolute cell count day 10 =* cell count day 10 * splitting factor day 3 * splitting factor day 5 * splitting factor day 7

*Mean Multiplication: e.g. day 3-5:* abs. cell count day 5 / abs. cell count day 3

Anti-Fas-Ligand antibody (Fas-L antibody) (#556371, BD^TM^) was added at day 0 of expansion (10 µg/ml) with a matched control of the same patient or donor. Feasibility of functional inhibition was evaluated by inhibiting recombinant Fas-L induced killing of Jurkat-Cells *in vitro* in the indicated dose (**Fig. S4**).

Recombinant Fas-L was purchased from Cell Signaling^TM^ (His6Fas Ligand, #5452, Cell Signaling). To induce differentiation via non-apoptotic Fas-L signaling, cells were incubated with 33ng/ml Fas-L^1^ for 7 days and subsequently analyzed by flow-cytometry. Cells were cultivated with same splitting schedule as described above with a matched control. Anti-His-antibody was used for crosslinking (10 µg/ml, R&D^TM^, MAB050). Relative cell growth on day 14 was tested for significant differences with unpaired t-test.

***Collection of conditioned medium and T-cell expansion***

After incubating PBCM for 24h, the non-adherent cells were collected and washed. To obtain conditioned medium (CM), the supernatant was saved after centrifugation. CM was stored in aliquots at -80 °C and thawed as needed. T-cells of healthy donors were activated and expanded in CM for 7 days.

***Production of GFP-expressing Lentivectors***

Lentivirus was produced as reported previously^2^. In brief, HEK293T cells were transfected with a 3-plasmid system. Transfection was performed with 12 μg pCMVΔR8.2 (addgene; Plasmid # 12263), 6 μg pHIT G (addgene; Plasmid # 8454) and 12 μg pRRLU6 CPPT pSK GFP. Plasmids were diluted in 62 μl of 2M CaCl_2_ in a final volume of 500 μl, and 2x HBS phosphate buffer was added without mixing. After incubation for 15 min the mixture was distributed on 2.5x10^6^ packaging cells per ml in 10 cm tissue plates and incubated overnight. Next day, the supernatant was discarded and fresh DMEM complete medium was added. After 24 h the supernatant was harvested, pooled, and concentrated by centrifugation in Amicon® Ultra-15 Centrifugal Filter Devices (3500xg, 4°C, ≈ 15 min). Each Filter was loaded with
15 ml supernatant, the time to desired concentration varied between the runs. The goal was at least 10fold concentration of the lentivectors. The concentrated supernatant was portioned to 500 µl aliquots and stored at -80 °C immediately.

***Flow Cytometry***

On day 0, cells were stained with commercially available antibodies against CD3, 4, 8, 27, 28, 45 (Beckman Coulter^TM^). To determine transduction efficacy, flow cytometry was performed in the biosafety laboratories at the Department of Molecular Gastroenterologic Oncology, Ruhr-University Bochum (BD^TM^ FACS Canto II).

For Low-Density-Lipoprotein-Receptor (LDL-R) detection, a fraction of activated T-cells was stained with anti-LDL-R antibody (R&D^TM^) and anti-CD3 (Beckman Coulter^TM^) at day 4. The mean of APC-signal was used for further analysis. The remaining T-cells were transduced with GFP-lentivirus. The results of LDL-R expression and transduction efficacy assessed by flow cytometry were correlated.

T-cell differentiation during cell expansion was assessed at day 12. Antibodies against CD3, CD45RA, CD62L, CCR7, CD27, CD28 (all Miltenyi BiotecTM) and CD4 (Santa CruzTM, Dal-las, Tx, US) were applied. Flow-cytometry data were analyzed with FlowJo v10.6.1.

***Gene expression analysis and data processing***

An amount of 100 ng of total RNA was hybridized to Agilent whole-genome expression mi-croarrays (Human GE 4x44K, v2 G4845A, AMADID 026652, Agilent TechnologiesTM, Santa Clara, CA, US). RNA labeling, hybridization, and washings were carried out according to manufacturer's instructions. Images of hybridized microarrays were acquired with a DNA mi-croarray scanner (Agilent G2505B) and features were extracted using the Agilent Feature Ex-traction image analysis software (AFE) version A.10.7.3.1 with default protocols and settings. The AFE algorithm generates a single intensity measure for each feature, referred to as the total gene signal (TGS), which was used for further data analyses using the GeneSpring GX software package version 14.9.1. AFE-TGS were normalized by the quantile method. Subsequently, data were filtered on normalized expression values.

For identification of differentially expressed genes, only entities with at least 2 out of the total number of samples and values within the selected cut-off (50^th^ percentile) were further includ-ed in the data analysis process. Using the GeneSpring GX software package version 14.9.1, differentially expressed genes were identified via moderated t-test. Finally, only mRNAs with a ≥ 2fold change in the microarray analyses were further considered. Gene set enrichment anal-ysis (V 4.0.3.) software was used for analysis of predefined gene sets.

***Digital-Droplet PCR***

Digital-Droplet PCR (ddPCR) was used for calculating the mean of viral copies per cell (GFP) in genomic DNA of the transduced T-cells. DNA was extracted from T-cells 5 days after transduction (QIAamp DNA mini Kit). DNA was extracted from T-cells 5 days after transduction (QIAamp DNA mini Kit). Primers and the probe were designed according to the sequence of the vector:

F 5’ CAGAAGAACGGCATCAAGGT3’

R 5’ TCGAGGACGGCAGCGTGCAGC 3’

P 5’ GGTGCTCAGGTAGTGGTTG 3’

Workflow was performed as previously described ^3^. Briefly, PCR-Mix (Primer/Probe-Mix, Supermix, EcoRI, DNA) was digested for 10 min at 37°C followed by PCR reaction (95°C 10 min, 40 cycles of 94°C 30 s and 60°C 30 s, followed by 98°C 10 min). Readout was performed on the QX200 droplet reader. The GFP-Probe was FAM labeled, Hex-labeled RPPH1 was used as housekeeper. Mean copy number was calculated by dividing GFP through RPPH1 calls (Ratio).

**Supplementary Figures**

**
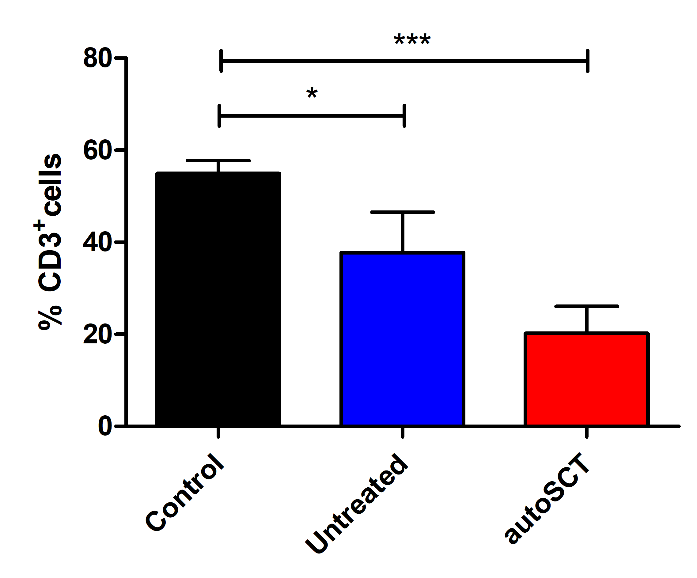
**

**Figure S1:**

Fraction (Mean +SEM) of CD3^+^ cells in buffy coats before cell expansion (control n=10, untreated n=5, autoSCT n=9).

**
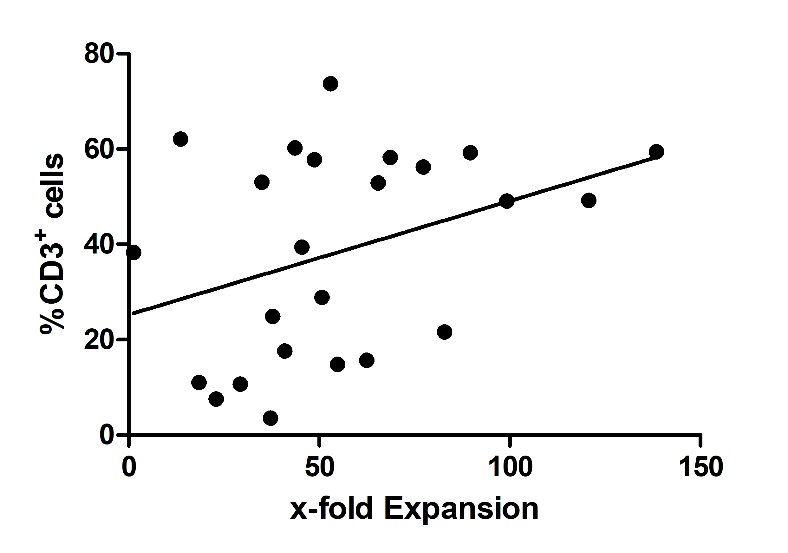
**

**Figure S2:**

Correlation of CD3^+^ cells and expansion in buffy coats before expansion (Day 0). Slope is not significantly different from zero (p=0.07), R^2^ = 0.13.

**

**

**Figure S3:**

T-cell phenotype at day 12 based on CCR7 and CD45RA expression (healthy n=5, untreated n=2, autoSCT n=5). In untreated patients, T_Eff_ were significantly enriched compared to patients after autoSCT (p=0.004). In contrast, the fraction of T_EM_ was reduced (p=0.010). For calculation of the p-value, t-test with Welch’s correction was applied. (Symbols and bars: * = p<0.05, ** = p<0.001, *** = p<0.0001; long colored bars: statistical comparison of fractions in column 1 vs. column 3, short colored bars: statistical comparison of fractions in column 2 vs. column 3)


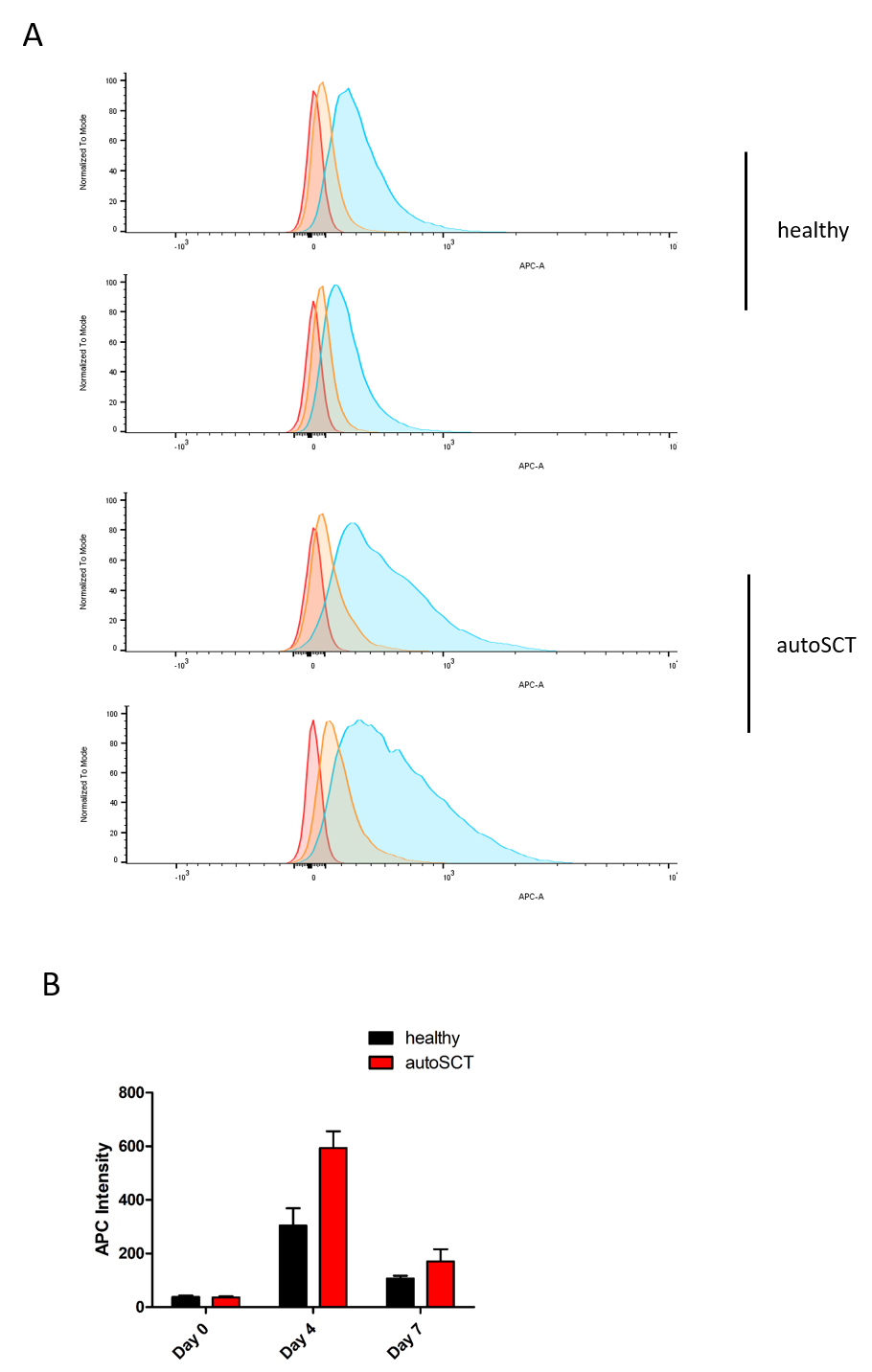


**Figure S4:**

**A:** Histograms of LDL-R expression as measured by APC in flow cytometry. LDL-Receptor expression (APC) is depicted on the x-axis, the y-axis was normalized to modal. Red: day 0, blue: day 4, yellow: day 7. Following expansion, expression of LDL is upregulated and subsequently downregulated during further expansion. **B:** Columns of mean APC intensity at the indicated days.


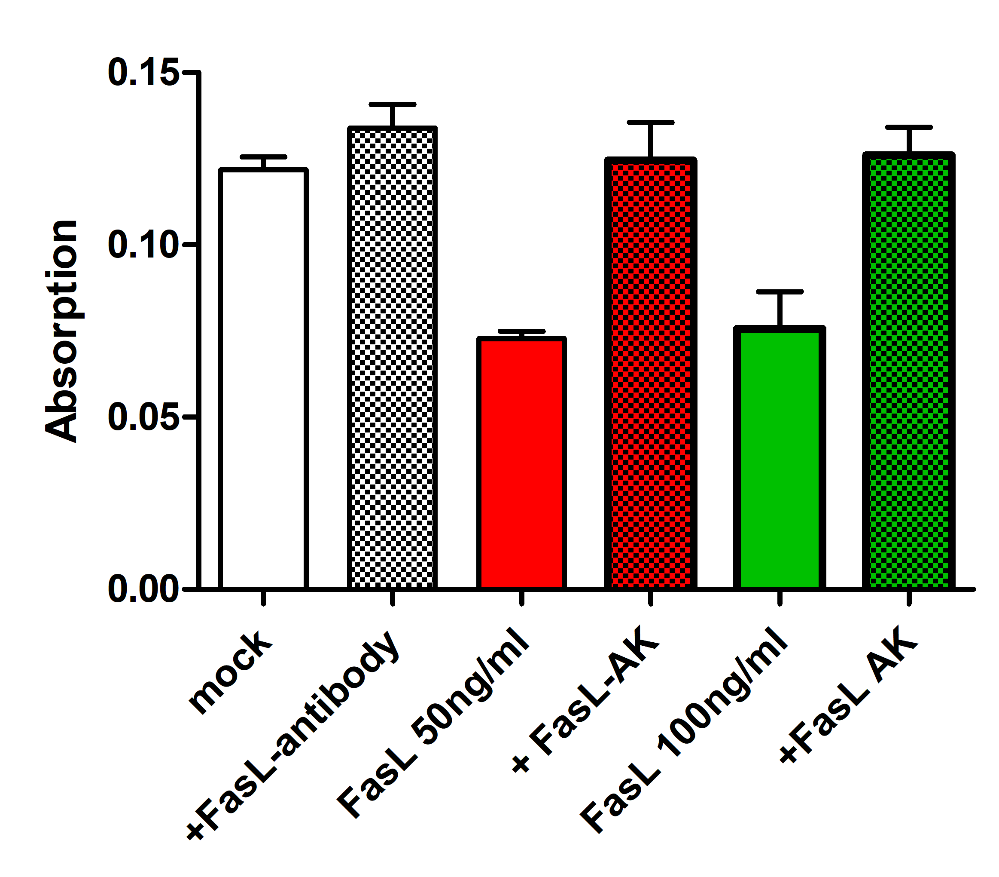


**Figure S5:**

Functional testing of Fas-Ligand antibody. Jurkat cells were seeded at a density of 10.000 cells/well in a 96-well plate in complete medium. Fas-Ligand was added at the indicated dose (His6Fas Ligand, #5452, Cell Signaling), anti-His-antibody (10µg/ml R&D^TM^) was added for crosslinking. Fas-Ligand antibody (10µg/ml) was added to mock, 50 ng/ml Fas-L and 100 ng/ml Fas-L assays. After 24h, XTT-assays (PromoCell) were performed. Incubation of XTT-reagent was 3h. Absorption was measured at 450 nm (630 nm reference). Higher absorption indicated a higher proportion of vital cells in the well. Fas-L antibody clearly inhibited apoptosis induced by Fas-Ligand. Columns shown are mean + SEM (triplicate measurements).

| Patient | Relative cell growth 3 months after autoSCT | Relative cell growth  follow-up | CD27^-^/28^-^ 3 months after autoSCT | CD27-/28- follow-up |
| --- | --- | --- | --- | --- |
| P7 | 29,29 | 16,93 | 55,2 | 65,7 |
| P8 | 13,38 | 19,76 | 73,4 | 59,3 |
| P14 | 13,66 | 27,39 | 85,5 | 72,4 |
| P16 | 18,46 | 21,64 | 56,1 | 18 |

**Table S1:** Detail information on 4 patients from **Figure 3 A**.

**References**

1. Klebanoff CA, Scott CD, Leonardi AJ, et al. Memory T cell-driven differentiation of naive cells impairs adoptive immunotherapy. J Clin Invest. 2016;126(1):318–34.

2. Baraniskin A, Birkenkamp-Demtroder K, Maghnouj A, et al. MiR-30a-5p suppresses tumor growth in colon carcinoma by targeting DTL. Carcinogenesis. 2012;33(4):732–9.

3. Mika T, Baraniskin A, Ladigan S, et al. Digital droplet PCR-based chimerism analysis for monitoring of hematopoietic engraftment after allogeneic stem cell transplantation. Int J Lab Hematol. 2019;41(5):615–21.
